# Identifying rare and common disease associated variants in genomic data using Parkinson’s disease as a model

**DOI:** 10.1186/s12929-014-0088-9

**Published:** 2014-08-30

**Authors:** Ying-Chao Lin, Ai-Ru Hsieh, Ching-Lin Hsiao, Shang-Jung Wu, Hui-Min Wang, Ie-Bin Lian, Cathy SJ Fann

**Affiliations:** Institute of Biomedical Informatics, National Yang-Ming University, Taipei, Taiwan; Bioinformatics Program, Taiwan International Graduate Program, Institute of Information Science, Academia Sinica, Taipei, Taiwan; Institute of Biomedical Sciences, Academia Sinica, Taipei, Taiwan; Graduate Institute of Biostatistics, China Medical University, Taichung, Taiwan; Graduate Institute of Statistics and Information Science, National Changhua University of Education, Changhua, Taiwan; Institute of Public Health, National Yang-Ming University, Taipei, Taiwan

**Keywords:** Genomic data, Parkinson’s disease, Rare variant association, Sequencing association study, Next generation association study, GWAS, SNCA, HAS2, Kremen1, Wnt signalling pathway

## Abstract

**Background:**

Genome-wide association studies have been successful in identifying common genetic variants for human diseases. However, much of the heritable variation associated with diseases such as Parkinson’s disease remains unknown suggesting that many more risk loci are yet to be identified. Rare variants have become important in disease association studies for explaining missing heritability. Methods for detecting this type of association require prior knowledge on candidate genes and combining variants within the region. These methods may suffer from power loss in situations with many neutral variants or causal variants with opposite effects.

**Results:**

We propose a method capable of scanning genetic variants to identify the region most likely harbouring disease gene with rare and/or common causal variants. Our method assigns a score at each individual variant based on our scoring system. It uses aggregate scores to identify the region with disease association. We evaluate performance by simulation based on 1000 Genomes sequencing data and compare with three commonly used methods. We use a Parkinson’s disease case–control dataset as a model to demonstrate the application of our method.

Our method has better power than CMC and WSS and similar power to SKAT-O with well-controlled type I error under simulation based on 1000 Genomes sequencing data. In real data analysis, we confirm the association of α-synuclein gene (SNCA) with Parkinson’s disease (p = 0.005). We further identify association with hyaluronan synthase 2 (HAS2, p = 0.028) and kringle containing transmembrane protein 1 (KREMEN1, p = 0.006). KREMEN1 is associated with Wnt signalling pathway which has been shown to play an important role for neurodegeneration in Parkinson’s disease.

**Conclusions:**

Our method is time efficient and less sensitive to inclusion of neutral variants and direction effect of causal variants. It can narrow down a genomic region or a chromosome to a disease associated region. Using Parkinson’s disease as a model, our method not only confirms association for a known gene but also identifies two genes previously found by other studies. In spite of many existing methods, we conclude that our method serves as an efficient alternative for exploring genomic data containing both rare and common variants.

**Electronic supplementary material:**

The online version of this article (doi:10.1186/s12929-014-0088-9) contains supplementary material, which is available to authorized users.

## Background

Genome-wide association studies (GWAS) have been successful in identifying common genetic variants underlying human diseases and complex phenotypes [1]. For most traits, however, the sum of the identified genetic effects generally comprises less than half of the estimated trait heritability [2]. For Parkinson’s disease, studies have shown that the genetic variance identified by top GWAS hits alone is between 3% to 5% and it is about 27% by meta-analysis [3]. Possible explanations for the missing heritability include low-frequency polymorphisms or rare variants that are not captured by genotyping platforms and undetected small effects from different loci that may together comprise a significant contribution to heritability [4,5]. Recent advances in next generation sequencing technology have made genetic variants over a wider frequency spectrum available for disease association studies. With efforts from the 1000 Genomes Project (TGP) which sought to identify most rare genetic variants in a group of 1092 multi-ethnic individuals, a new generation of GWAS is being designed to enable the discovery of lower frequency genetic variants by using next generation sequencing data [6,7].

Rare variants, here defined as genetic variants with minor allele frequencies (MAFs) less than 1%, can play an important role in the etiology of complex traits [8]. Traditional single-variant test focuses on the marginal effect on disease by analyzing markers one at a time. The power of this type of test is low for rare variant association due to the inherent low frequency and large number of rare variants in the genome [9,10] particularly in large scale data such as sequencing based association studies. It has been suggested that pooling minor alleles at rare variants into a measure of burden at a locus can help enrich association signals and mitigate the power loss due to allelic heterogeneity and high dimensionality [11]. Pooling based on either collapsing rare variants into a single indicator of the presence of any minor alleles or summing weighted minor allele counts over rare and/or common variants has been the earliest proposal for detecting rare variant association [12]. For instance, the Combined Multivariate and Collapsing (CMC) method and the Weighted Sum Statistic (WSS) method have been shown to be superior to single-variant test statistics [13]. CMC collapses genotypes across all variants into an indicator variable such that an individual is coded as 1 if a rare allele as defined by the pre-specified MAF threshold is present at any of the variant sites, and as 0 otherwise, followed by a multivariate test such as Hotelling’s T^2^ test [14]. WSS uses a non-parametric sum test in which each mutation is weighted according to its count in control subjects and then permutes the disease phenotype to assess the significance of a Wilcoxon-type test statistic [15].

In the case when neutral variants are included, as is likely to be so in sequencing based association data where most variants are expected to have little or no effect, pooling tests lose power. Such tests lose power especially when the extent of negative linkage disequilibrium (i.e., D < 0) between neutral and risk variants is large thereby causing masking of the true disease association attributed to rare variant effect [16]. The inclusion of protective variants whose effects are in the opposite direction from risk variants will further exacerbate the masking effect and power loss in finding the susceptibility gene [17]. Excluding variants based on prior information such as annotation and functional predictions may improve the performance of pooling tests, however, it may result in loss of information due to bias in prior knowledge [18].

To circumvent the masking effect of non-causal and protective variants, alternative methods have been devised using approaches such as model selection, adaptive MAF thresholds and/or weighting schemes to determine how and which variants to collapse in order to improve power [12,19-27]. An example is the variable-threshold method which assumes that the effects of the combined rare mutations on the phenotype are in the same direction. It uses the maximum of the test statistics over all allele frequency thresholds to assess statistical significance by permutation [21]. To detect both deleterious and protective effects, several methods such as adaptive sum test and burden or mutation position test have incorporated the signs of the observed effects into the burden test statistics [22,23].

Sums of single-variant test statistics have previously been shown to be powerful for joint inference in association studies [24-26]. Multi-variant and gene- or region-based methods that make joint inference based on single-variant test statistics consider only the magnitude of deviation from the expected null distribution. These type of methods are inherently robust to the inclusion of neutral and protective variants [13,28]. A recent study has pointed out that rare variant association tests (e.g., SKAT) that are robust to the inclusion of neutral and protective variants have actually arrived at procedures similar to performing region-wise inference using single-variant test statistics [16,27,29,30]. This type of tests provides an overall result without identifying the region with disease association.

In this study, we propose a method that is capable of scanning genetic variants and identifies the sub-sequence with disease association. Our method utilizes single-variant association tests to generate single-variant scores at each individual marker and rather than summing the test statistics, we sum the single-variant scores derived by our scoring system to generate the partial sum scores along the sequence. To reduce dimensionality which has been a challenging problem with large-scale data such as in sequencing-based association studies, our method uses a partitioning procedure to divide the sequence into sub-sequences and test the maximal region-wise score for the identification of the disease gene by permutation. We compare our method to the two originally proposed pooling tests (i.e., CMC [14] and WSS [15]) and the optimal test of SKAT, namely, SKAT-O [29], under several simulation scenarios. The simulation datasets have been generated based on sequencing data of the European reference panel from TGP [6]. Using Parkinson’s disease as a model, we demonstrate the application of our method by analyzing a real case–control dataset for Parkinson’s disease [31].

## Methods

### General maximal segmental score (GMSS)

Our method is based on a more general class of maximal segmental scores (GMSS), with the conventional maximal segmental scores (MSS) as its special case [32]. The MSS has wide applications on measuring the sequence similarity and has been implemented in algorithms such as the Basic Local Alignment Search Tool (BLAST) [33,34]. When comparing two amino acid query sequences based on pairs of corresponding nucleotides, BLAST often uses a discrete score system by scoring a matched pair with 1, a non-matched with −1, and gaps (insertion or deletion) with certain negative values as penalty [34].

In this study which aims to locate the disease gene, the idea of GMSS is to identify a region with the strongest “composite” signal of case–control difference. The significance of the difference is measured by the p-value from each marker within the region. Different from the discrete nature of matching-vs.-non-matching status for nucleotide or protein pairs considered in BLAST, the p-values here are continuous. Thus, to be able to derive the asymptotic distribution of the maximal segmental scores for testing purpose, our pervious methods categorize the p-values into discrete scores by using scoring systems similar to those used in BLAST [35,36]. However, if a complete case–control data set and efficient computation capacity are available, using a more general continuous score is plausible. We propose to use a different scoring system with continuous scores in this study and the scoring system is described in the following section.

The hypothesis that we consider in this study concerns sequence data of *n* markers from a case–control sample for association testing. The null hypothesis (*H*_0_) is that there is no disease gene in the sequence of markers vs. the alternative (*H*_1_) that there is a disease gene in the sequence. We propose to utilize p-values from single-variant association tests to generate the single-variant score at each individual marker along the sequence. Although our method is not limited by the type of association tests used to derive p-values, here we consider the single-variant Cochran-Armitage trend chi-square test for its robustness to the direction of the effect and departures from Hardy-Weinberg equilibrium, ease of calculation and wide applicability [37]. As a general version of the maximal segmental scores for disease association studies [35], we describe the GMSS method in the following four sections. A potentially useful resource resulting from this work is a freely available R code posted on the following website http://www.csjfann.ibms.sinica.edu.tw/eag/programlist/GMSS/GMSS.html. No experimental research is reported in this manuscript.

### Calculating single-variant scores and partial sums

We propose a scoring system for converting the p-value into a score at each marker in the sequence. Given a sequence of p-values {*p*_1,…,_*p*_*n*_} each derived from the Cochran-Armitage trend Chi-square test, the single-variant score *Y*_*i*_ at marker *i* is defined as ${Y}_i=-2 \ln \left({p}_i\right)-\left[{\chi}_{1-\alpha}^2(4)-{\chi}_{1-\alpha}^2(2)\right]$, *i = 1,…,n*. The scoring system is derived based on Fisher’s p-value combining method [38].

The single-variant score sequence {*Y*_1,…,_*Y*_*n*_} is calculated by setting the alpha at a conventional level of 5%. Thus, the equation for the single-variant scores becomes *Y*_*i*_ = −2ln(*p*_1_)-3.5, *i = 1,…,n*. In an attempt to locate a segment with disease association when scanning a marker sequence, the reduction of 3.5 used in our scoring equation is considered as the token to pay when including an adjoining marker. For a single marker, the threshold of significance at 5% level would be ${\chi}_{0.95}^2(2)=5.99$ with the degrees of freedom (df) of 2. When an additional marker is considered, the df would become 4 and the threshold of significance would be increased to ${\chi}_{0.95}^2(4)=9.49$. Thus, when including an adjoining marker, the threshold of significance would be elevated by 3.5 (i.e. from 5.99 to 9.49), according to Fisher’s method [38]. Based on this observation that the threshold of significance would be elevated by 3.5, we take this into account when calculating the scores by including a payoff of 3.5. Our scoring equation is a combination of a chi-squared statistic and a payoff value of 3.5 for converting p-values to continuous scores. Note that this is the inherent level on a marker test based on Chi-squares for constructing the empirical distribution of the null hypothesis, not directly related to the level of significance for the test on the region based on the general maximal segmental score to be described later.

The single-variant scores {*Y*_1,…,_*Y*_*n*_} are then aggregated in a sequential fashion to form a sequence of partial sum scores {*U*_0_,*U*_1_…,*U*_n_} where ${U}_0=0,\kern0.5em {U}_{\mathrm{m}}={\displaystyle {\sum}_{i=1}^m{Y}_i,\kern0.5em \mathrm{m}=1,2,\dots },n$. *U*_*0*_ is the initial partial sum score. *U*_*m*_ is the partial sum up to the *m*^*th*^ marker in the sequence. The partial sum scores *U*_*m*_ as a function of the single-variant scores *Y*_*i*_ are not monotonically non-decreasing since the *Yi*’s can have negative values.

### Identifying ladder points and sub-sequences

**Figure 1:**
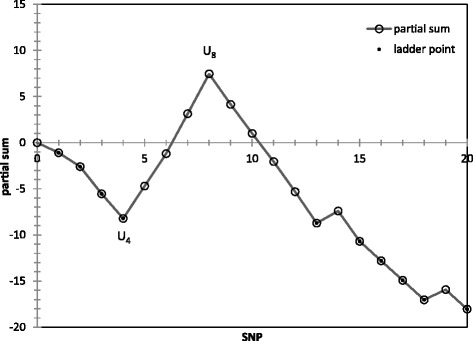
**An example of partial sum scores for 20 SNPs.** Ladder points (solid dots) are the positions with record new lows of the partial sum scores (open circles). U8 and U4 indicate the partial sum scores for the 8th and 4th SNPs, respectively. The general maximal segmental score is the difference between U8 and U4 which occurs in the sub-sequence bounded by the 4th and 13th SNPs.

Given the partial sum score sequence {*U*_0_,*U*_1_…,*U*_n_}, the ladder points are defined as ${J}_0=0,\kern0.5em {J}_v={\displaystyle \underset{j}{ \min }}\left\{j:j\ge {J}_{v-1}+1,\ {U}_j-{U}_{J_{v-1}}<0\right\},\kern0.5em \nu =1,2,\dots$ [32]. Figure 1 shows an example of the ladder points (solid dots) for a sequence of 20 SNPs. Ladder points are where new record lows of the partial sum scores occur as one moves along the sequence marker by marker. As the ladder points are identified along the sequence, regions bounded by two adjacent ladder points form sub-sequences. As shown in Figure 1, there are ten ladder points which form ten sub-sequences. The ladder points are the 1st to 4th, 13th, 15th to 18th, and 20th SNPs with record low partial sums U_1_ to U_4_, U_13_, U_15_ to U_18_, and U_20_, respectively. Note that a ladder point occurs only when the partial sum is lower than the partial sum of the last ladder point.

The way to which we select the ladder points is motivated by methods for matching amino acid sequences based on similarity scores such as BLAST [32-34]. For our disease association study, the purpose for the ladder points is to form sub-sequences along the input marker sequence so that within each smaller region, we can calculate the general segmental score in the next section. By using the ladder points, it helps us to find the region with the highest score which is likely to be where disease association would be identified. This also helps to reduce data dimensionality confronted by high-throughput data.

### Calculating general segmental scores and the general maximal segmental score

For each sub-sequence, a general segmental score i.e., *S*_*v*_ is calculated as the difference between the largest partial sum within a sub-sequence and the initial partial sum value of the sub-sequence. The equation used to obtain the segmental score is shown below.

$$ {S}_v={\displaystyle \underset{J_{v-1}\le j<{J}_v}{ \max }}\left({U}_j-{U}_{J_{v-1}}\right),\kern0.5em \nu =1,2,\dots . $$

The general segmental scores are non-negative values defined as the uphill height within the sub-sequence bounded by two adjacent ladder points. In the example shown in Figure 1, the general segmental scores for the ten sub-sequences are 0, 0, 0, 0, (U_8_-U_4_), (U_14_-U_13_), 0, 0, 0, (U_19_-U_18_), respectively. The zero score indicates no increase in-between the two adjacent ladder points. The larger the score is, the higher the likeliness for the existence of target SNPs to be within the segment.

The largest segmental score of the whole sequence is identified as the general maximal segmental score *GMSS* = max{*S*_1_,*S*_2_,…}. The GMSS is (U_8_-U_4_) in the example shown in Figure 1. The significance of *GMSS* is evaluated via permutation as shown below.

### Permutation for empirical null distribution

To test the hypothesis of disease gene, the p-value of *GMSS* is estimated by permutation. To derive the empirical distribution of *GMSS* while preserving the structure of the genotype data, we perform 1,000 replications by shuffling the case–control status followed by steps described above each time. The observed *GMSS* is then compared to the empirical distribution to derive the empirical p-value which is defined as the proportion of permuted *GMSS* that are at least as extreme as the observed one under the empirical distribution. Our rejection rule is to reject the null hypothesis if *GMSS* > *c*, where *P*(*GMSS* > *c|H*_0_) = α and *c* is the threshold corresponding to the level of significance α = 0.05 under the null hypothesis *H*_0_.

### Simulation studies

We conducted simulation studies by using HAPGEN2 [39] which generates case–control genotypes conditional on a reference set of population haplotypes and an estimate of the fine-scale recombination rate across the region. To consider more realistic set-ups, our simulation was based on version 3 of the Phase 1 integrated data of TGP released in March of 2012 [6]. We retained single nucleotide polymorphisms (SNPs) in the reference panel of 379 Europeans (EUR) [40] from TGP for the simulation. Using this reference dataset, we simulated case–control data with and without the disease gene under different scenarios. Each of the scenarios was performed with 200 replications in our study with the following setups.

### Simulation without disease gene

We chose a 1 MB region on chromosome (chr) 4 with 5086 SNPs and simulated data without the disease gene for the sample size of 5000 cases and 5000 controls [15,23,41].

### Simulation with disease gene

We generated case–control data by considering four factors that may influence the performance on detecting disease association, including (i) number of non-causal variants, (ii) number of rare causal variants in the disease gene, (iii) relative risk of rare causal variants, and (iv) combinations of rare and common causal variants in the disease gene. We considered two different numbers of non-causal variants (region sizes), two different numbers of rare causal variants and three different levels of relative risks. In addition, we simulated two scenarios for combinations of rare and common causal variants. In total, we simulated 14 different scenarios involving a mixture of rare and common causal variants.

For the number of non-causal variants, our simulation considered two different region sizes, namely, 1 MB and 0.1 MB, which corresponded to 5086 and 524 non-causal SNPs in TGP sequencing data. The smaller region i.e., 0.1 MB was representative of a larger human protein coding gene based on recent data (mean size: 27 kb, range: 1 kb–2400 kb) [42]. We assumed that one disease gene was embedded in the simulation region and the disease variants were located near the center of the marker sequence.

To determine the number of rare and common SNPs in the disease gene, we examined the distribution of rare variants (MAF < 1%) based on 1174 genes of chr 4 in the reference data. We found that the average proportion and number of rare variants were 41% and 115, respectively, which would correspond to a gene with 280 variants. Based on this observation, we simulated the disease gene with similar numbers of total and rare variants i.e., one that contained 280 SNPs where 130 SNPs were rare and the remaining 150 SNPs were common.

To investigate the effect of the number of rare causal variants in the disease gene, our simulation assumed that there were 50 and 70 rare causal variants. Re-sequencing studies of the coding parts of the human genes had suggested 50 disease variants to be a realistic level [15]. Some studies had assumed 50 rare causal variants in their simulations [15,16] and others had considered a range of 10% to 50% of rare variants to be causal [27,29]. Therefore, in our simulations, we considered scenarios with 50 and 70 rare causal variants which corresponded to about 38% and 54%, respectively, of the rare variants in the disease gene being causal.

To study the effect of relative risks (rr), the rare causal variants were assumed to have rr of 1.5, 1.7 and 2 [16]. The causal variants were arbitrarily chosen from the rare variants in the disease gene and an additive disease model was assumed. To study the impact of combinations of both rare and common causal variants, we considered two simulation scenarios. The scenarios assumed a combination of 50 rare causal variants with rr of 1.5 and one common causal variant with MAF of 10% and rr of 1.1 and 1.2 [30,43].

### Performance evaluation

We compared GMSS to CMC [14] and WSS [15] by examining the type I error rate and power in our simulation. We also compared to SKAT by using the unified approach with the option that improves the tail probability i.e., SKAT-O [29]. The MAF threshold for rare variants was 1% and default values were applied in the other parameters for CMC, WSS and SKAT-O for the analyses. Significance was declared at the p-value cut-off of 0.05.

For type I error analysis, we applied GMSS, CMC, WSS and SKAT-O to the simulation data without the disease gene. The type I error rate was defined as the proportion of replicates that were falsely declared as significant in the simulation without the disease gene.

For the analysis on power, we applied all four methods to the simulation data with disease gene. For CMC, WSS and SKAT-O, power was defined as the proportion of replicates which were declared significant in scenarios simulated with the disease gene. For GMSS, the power was defined as the proportion of replicates which yielded significant p-values and correctly identified the region of the true causal gene within the simulated marker sequence.

## Results

### Reference dataset

The reference dataset used in our simulation was from TGP sequencing data of 379 Europeans. The MAF distribution of 5086 markers in a 1 MB region in this reference panel was shown in Table 1. When using 1% as the MAF threshold for defining rare variants, our result showed that there were about 40% rare variants and 60% common variants in the reference data. The median and mean MAFs were 2.4% and 10.3%, respectively, in this dataset. The result on the MAF distribution based on all markers on chr 4 for the reference panel was similar (not shown). For the rare causal disease markers that were chosen arbitrarily for the simulation, the median MAF of the rare causal variants was 0.5% with the range being from 0.4% to 0.92% in the reference panel.

**Table 1:** **Minor allele frequency (MAF) distribution in a 1 MB region for the reference panel**

**MAF**	**< 1%**	**(1%, 2.4%)**	**(2.4%, 5%)**	**> 5%**
Proportion (%)	40	10	10	40

There are 5086 SNPs and the reference panel is 379 EUR in TGP.

### Type I error

For type I error analysis, we applied GMSS, CMC, WSS and SKAT-O to the simulation data with no disease gene. Table 2 showed the type I error rate over 200 replicates for these four methods under the simulation scenario with no disease gene in a 1 MB interval containing 5086 polymorphic SNPs in TGP sequencing data for 5000 cases and 5000 controls. The type I error rate was the lowest for GMSS (0.04) followed by CMC (0.05), SKAT-O (0.06) and WSS (0.08). The result indicated that all four methods had reasonably well-controlled type I error rate in our simulation.

**Table 2:** **Type I error rate under the simulation scenario of no disease gene**

**Method**	**GMSS**	**CMC**	**WSS**	**SKAT-O**
Type I error rate	0.04	0.05	0.08	0.06

Data were generated for 5000 cases and 5000 controls based on a 1 MB region (5086 SNPs) of TGP sequencing data.

### Power

**Figure 2:**
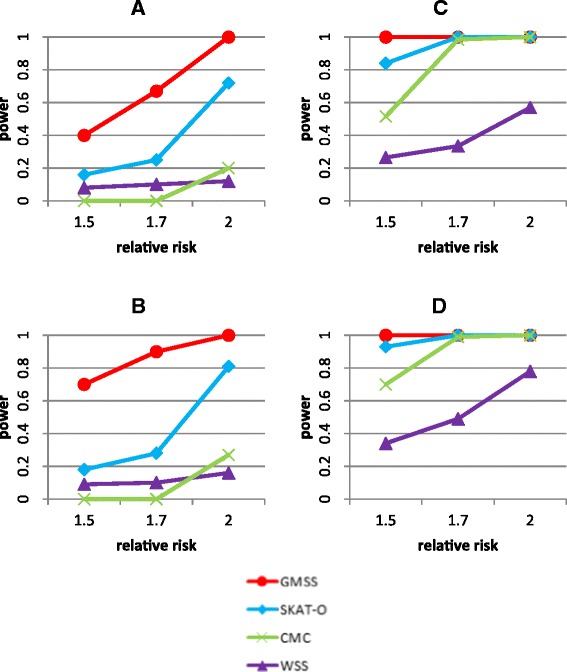
**Power for detecting the disease gene (280 SNPs) for 5000 cases and 5000 controls. A**. 50 rare causal variants (MAF < 1%) in a 1 MB (5086 non-causal SNPs) region of TGP sequencing data with relative risk (rr) of 1.5, 1.7 and 2. **B**. 70 rare causal variants (MAF < 1%) in a 1 MB (5086 non-causal SNPs) region of TGP sequencing data with rr of 1.5, 1.7 and 2. **C**. 50 rare causal variants in a 0.1 MB (524 non-causal SNPs) region of TGP sequencing data with rr of 1.5, 1.7 and 2. **D**. 70 rare causal variants in a 0.1 MB (524 non-causal SNPs) region of TGP sequencing data with rr of 1.5, 1.7 and 2.

For comparing the performance on the power for detecting the disease gene, we applied GMSS, CMC, WSS and SKAT-O to the simulation data with disease gene. Figure 2 showed the results on power of these methods for the impact of non-causal variants, rare causal variants and relative risk (rr).

In scenarios where there were 50 rare causal variants in a sequence with 5086 non-causal variants (1 MB), the power for detecting the disease gene increased for all four methods as rr increased with the rank in power being GMSS > SKAT-O > WSS > CMC (Panel A). Exceptions were when rr was raised to 2, CMC had higher power than WSS. For CMC, WSS and SKAT-O, the results on the significance for disease association were for the overall region in the analysis while GMSS provided not only the p-value but also the location of a finer region for the identified disease association. The results on the regions identified by GMSS under these simulation scenarios showed that the region with association found by GMSS spanned the disease gene and an average of 27, 15 and 15 SNPs that were assumed non-causal when rr was 1.5, 1.7 and 2, respectively. The same trend was observed for the power with 70 rare causal variants (Panel B). In these scenarios, the regions identified by GMSS overlapped with the disease gene and an additional 36, 35 and 34 non-causal SNPs on average for rr of 1.5, 1.7 and 2, respectively.

When the number of non-causal variants was smaller (i.e., 524 variants in 0.1 MB), the power for detecting the disease gene was increased for all four methods across all levels of rr in the scenarios with 50 rare causal variants (Panel C). The rank in power was GMSS > SKAT-O > CMC > WSS. The regions identified by GMSS spanned the disease gene and an average of 9, 12 and 10 non-causal SNPs for rr of 1.5, 1.7 and 2, respectively. Our results showed the same trend in power when the number of rare causal variants was increased to 70 (Panel D). In these scenarios, the regions identified by GMSS overlapped with the disease gene and an additional 9, 9 and 10 non-causal SNPs on average for rr of 1.5, 1.7 and 2, respectively.

For the impact of relative risk, the overall result showed that there was an increasing trend in power as rr increased for all methods. The average power was increased by 44%, 46%, 30% and 14% for CMC, WSS, SKAT-O and GMSS, respectively, as the level of rr increased from 1.5 to 1.7 and 2.

For the impact of non-causal variants, there was an increasing trend in power for all the methods when the number of non-causal variants decreased. There was an average of 3.1-fold, 2.4-fold and less than 1-fold increase in power for WSS, SKAT-O and GMSS, respectively as the number of non-causal variants decreased from 5086 (1 MB) to 524 SNPs (0.1 MB). For CMC, there was a 10-fold increase in the average power when the number of non-causal variants decreased in the simulation.

For the impact of number of rare causal variants, the average increase in power going from 50 to 70 rare causal SNPs was about 11%, 15%, 12% and 36% for CMC, WSS, SKAT-O and GMSS, respectively, in the 1 MB scenarios. A similar trend was also shown in the 0.1 MB settings. Although both the number of rare causal variants and non-causal variants affected the power for all the methods, some methods were more sensitive to one than the other. For example, the results appeared to show that SKAT-O was relatively less sensitive to the number of rare causal variants and GMSS was relatively less sensitive to the number of neutral markers in the sequence.

To study the effect of both rare and common causal variants, we compared the power of CMC, WSS, SKAT-O and GMSS in scenarios with combinations of these variants. In the scenario that assumed 50 rare causal variants with rr of 1.5 and one common causal variant with MAF of about 10% and rr of 1.1 in a 1 MB interval of TGP sequencing data, the power of GMSS for detecting the disease gene was 0.52 and the identified region overlapped with the disease gene and an average of 117 additional non-causal SNPs. When the rr for the common causal variant was raised to 1.2, the power of GMSS was increased to 0.65 and the identified region overlapped with the disease gene and an average of 23 additional non-causal SNPs. The results in the combination scenarios for CMC, WSS and SKAT-O showed almost no difference to that from the scenario with 50 rare causal variants with rr of 1.5.

### Application of GMSS - using Parkinson’s disease as a model

To demonstrate the application of GMSS on real data, we performed a real data analysis using a publically available Parkinson’s disease dataset from dbGAP (CIDR: Payami Omni GWA Project, Accession: phs000196.v2.p1) [31,44]. The downloaded dataset contained IlluminaHumanOmni1-Quad_v1-0_B GWAS data for 2000 individuals with Parkinson’s disease and 1986 control subjects [31]. To obtain the single-variant score sequence, we performed Cochran-Armitage trend chi-square test by using PLINK to obtain the p-value at each individual SNP [45]. We followed the steps as described in method to perform association analysis using GMSS as well as SKAT-O [29] and compared the results with the current literature.

Parkinson’s disease has been one of the most common neurodegenerative diseases affecting over 1% of the elderly population [46]. Many studies have provided evidence on the association between Parkinson’s disease and the SNCA gene on chromosome 4 [31,46-49]. To ensure that our method could at least identify the association of this gene, we analyzed all 59,377 SNPs on chr 4 to identify disease association. The result from our analysis showed that there was a significant disease association in a region of 706.9 KB consisting of 155 SNPs (Additional file 1) (p-value = 0.033). About 14% of the SNPs in this region had MAFs below 1% which was similar to the distribution observed when using all SNPs on chr 4 in this dataset. The identified region included the SNCA locus which was a protein coding gene that may serve to integrate presynaptic signalling and membrane trafficking and had previously been implicated in the pathogenesis of diseases such as Parkinson’s and Alzheimer’s diseases [31,46-49].

In comparison, SKAT-O could not be directly applied on the data due to the large number of markers. We thus truncated the data by removing markers on both ends and analyzed a sequence of a smaller set of markers (30,000 SNPs) by using both GMSS and SKAT-O. Our results indicated that GMSS identified the same sub-region for disease association as when the entire chromosome was analyzed (p = 0.005). The result for SKAT-O based on these 30,000 SNPs was also significant (p = 0.001) albeit with no further indication on the corresponding region for the association on chr 4. This analysis by GMSS took about 10 minutes using a linux workstation with 2 GHz CPU and 128G RAM while the analysis by SKAT-O took more than 72 hours with the same computing power.

The hyaluronan synthase 2 (HAS2) gene on chr 8 was not significant in the main sample or meta-analysis, but it was marginally significant in a replication cohort [31]. Based on this, as well as independent evidence showing association for this gene [50], we analyzed this region to see if our method is capable of identifying the association in the main sample. We performed the analysis by using GMSS and compared our result with SKAT-O. The analysis by GMSS identified a region of 22 SNPs (Additional file 1) with significant disease association (p = 0.028). About 9% of the SNPs in this region had MAFs below 1% in this dataset. The result by SKAT-O identified no significant association for this gene (p = 0.56).

Recently, the WNT signaling pathway involving KREMEN1 has been reported to be associated with the cell replacement therapy for Parkinson’s disease [51]. Evidence has indicated that knockdown of Kremen1 significantly enhance axon outgrowh in murine dorsal root ganglion neuronal culturals [52]. KREMEN1 has been shown to be a negative regulator of the canonical WNT signaling pathway that is important for the healthy functioning of the adult brain [53]. A potential role for this gene is in the regulation of cellular responses upon extracellular stimulus or cell-cell interaction in neuronal and/or muscle cells [54]. This gene was previously reported to have an association with schizophrenia [55]. To test the association between KREMEN1 and Parkinson’s disease, we performed GMSS using 225 SNPs spanning 1 MB on chr 22. The result identified a 63.7 KB region of 25 SNPs (Additional file 1) with significant disease association (p-value = 0.006). About 9% of these SNPs were rare (MAFs below 1%). The association was not identified when using SKAT-O (p = 0.2) and was not reported in the original study [31].

## Discussion

With growing marker density and amounts of rare variants available in sequencing data, statistical methods have been developed to address the issue of missing heritability in identifying the causal variants for common diseases. Due to the large number of small individual effect and low allele frequency of rare variants, traditional single-variant tests have low power in detecting rare variant association. It has been shown that rather than analyzing rare variants individually, aggregating the variants together can help mitigate the problem.

In this study, we propose a GMSS method that utilizes the aggregate scores from markers within a neighborhood to identify the region most likely harbouring the disease gene with rare and/or common causal variants. We develop a scoring system that is a combination of a chi-squared statistic and a payoff value following the concept of Fisher’s p-value combining approach [38]. Our scoring system assigns continuous scores for individual markers based on single-variant association p-values. The scores are aggregated sequentially and a region-partitioning procedure identifies the sub-sequence that has the strongest combined evidence for disease association. Unlike region- or gene-based methods which require prior information on candidate regions, our method is capable of identifying the segment with disease association. For the methods that we have compared in this study, they would require prior knowledge as to the region or candidate gene to associate it with disease. This is because these methods provide an overall p-value for disease association for the entire input data sequence. In situations when the candidate region is not known or when an entire chromosome is analyzed by these methods, their result would not be able to guide researchers to identify a finer segment.

Our simulation is based on the TGP sequencing data of the EUR reference panel in order to mimic realistic minor allele frequencies and linkage disequilibrium spectra [6,39,40]. We simulate case–control datasets under different scenarios to evaluate the type I error rate and power by comparing GMSS with CMC [14], WSS [15] and SKAT-O [29]. The factors that are examined include the number of non-causal variants, the number of rare causal variants, relative risk and combinations of rare and common causal variants. Under all the simulation settings considered in this study, the results show that GMSS has similar or better power with well-maintained type I error rate as compared with the other methods.

Our results indicate that the number of rare causal variants and level of relative risk have a positive effect while the number of non-causal variants has a negative effect on power for all these methods. While the methods that we compare with in this study are affected by these factors, some methods are relatively more sensitive to one than another. For instance, CMC and WSS are relatively more sensitive to number of non-causal variants and relative risk in our simulations. Both CMC and WSS pool the variants together for detecting rare variant association. CMC pools variants under the MAF threshold together but it loses power when the regions are large due to the increased degrees of freedom for the Hotelling’s T^2^ test. While results for this method might be different when altering the pre-set MAF threshold, GMSS requires no such threshold to be specified in advance. For WSS, the variants are weighted according to their frequencies by allowing rare variants to contribute more to the test statistic. These type of methods have an advantage when all the variants that are rare are causal and with the same direction effect on the disease trait, scenarios unlikely to be observed with large-scale data such as in sequencing based association studies. Our method utilizes scores based on p-values for combining the variants and thus is not affected by the direction of the effect of the disease variants.

It has been suggested that methods that use sums of single-variant statistics such as SKAT-O have better power for detecting rare variant association as compared with pooling tests [16]. As shown in our result, SKAT-O has a better power than pooling tests in general. It is a linear combination of pooling test and SKAT, which is a multivariate technique using generalized linear models. SKAT-O optimizes between pooling test and SKAT to achieve more power. Although our result shows that the change in power when increasing the number of rare causal variants is not as substantial for SKAT-O as compared with our method, the power is increased by a larger amount than GMSS when the number of neutral variants decreases in our simulation scenarios.

The performance in power for GMSS is better than SKAT-O in the scenarios with smaller relative risk and more neutral variants. In scenarios with smaller regions and fewer neutral variants, the power for GMSS is similar to SKAT-O. Our simulation result shows that the number of rare causal variants has a relatively larger impact on GMSS than the other methods. When increasing the number of rare causal variants, the increase in power for GMSS is the greatest among these methods. When there are many markers involved in the data such as in sequencing based association studies, our results show that GMSS is less sensitive to the inclusion of neutral variants but relatively more sensitive to the number of rare causal variants. In our study, we assume there are 50 and 70 rare causal variants since it has been suggested to be a realistic number of disease variants in re-sequencing studies of the coding regions of the human genes [15,16]. We assume that one disease gene is embedded in the simulation region and the disease variants are located near the center of the marker sequence. One of the limitations of the proposed method is that it is sensitive to the clustering of disease variants and the designed simulation studies. Further studies are required to investigate situations with different clustering patterns of the disease gene.

While the other methods only provide a p-value for indicating whether somewhere along the sequence there is disease association, GMSS is able to identify finer segments within the sequence that shows evidence for disease association. Our results indicate that the segments identified by GMSS contain the disease gene plus around 9 to 117 additional non-causal variants on average. The region identified by GMSS would not always be within the same absolute size range. By using the simulation study, we aim to show that this method was capable of identifying the segment harbouring the underlying disease gene, a feature that would be an advantage for exploratory analysis on long sequence data in association studies.

One of the aims of this study is to identify the disease susceptibility region in a long sequence of p-values. Therefore, we use more stringent criteria for the power simulations than for other tests that we compare with in this study. Under the simulation scenario of a 1 MB region, the results show that the proportion of significant wrong hits ranges from 0.01 to 0.07; it is slightly higher when the number of causal variants and level of relative risk are lower.

Since it is likely that rare and common variants both contribute to disease association in actual data, our simulation also examine the scenarios with combinations of both types of variants. The result shows that the power for GMSS becomes higher than the scenario with only rare causal variants, suggesting that our method is able to exploit the information from all variants. The power for CMC, WSS and SKAT-O is almost the same as the scenarios without the common causal variant while the power for GMSS is increased in the combination scenarios. For pooling tests such as CMC and WSS, the effect of the common causal variant seems to be diluted due to their pooling and weighting schemes that favor rare variants. Our simulation assumes 50 rare causal variants (rr = 1.5) and one common causal variant with weak effect (rr = 1.1 and 1.2) in a 1 MB region. Possible reasons that the power for SKAT-O remains similar could be that the number and effect of the common causal variant might be too small and it might require a much larger contribution to disease association from both types of variants to overcome the effect of neutral variants. Overall, when the effect and number of common causal variant are small, GMSS does not ignore this information. Under the scenario where both common and rare variants contribute to the disease risk, GMSS would be able to use information from both types of variants thereby enhancing the power for identifying disease association. We also use the most recent development of SKAT developed to deal with the combined effect of both common and rare causal variants [30]. The results are similar (not shown) to SKAT-O in the scenarios presented in this study.

For GMSS, we analyze the scenario that has only the common causal variant with rr of 1.1 by excluding the rare causal variants. Excluding variants based on MAF threshold has been a standard practice in traditional GWAS. Under this scenario, the power for detecting the disease gene is very low (power = 0.03) indicating that the inclusion of both rare and common causal variants might yield better power for identifying disease gene association. This finding suggests that including rare variants in the analysis might in part explain the missing heritability problem.

Our method deals with a sequence of p-values. For sequencing data such as exomes, our method would not be able to analyze the raw data. However, in a case–control or family study design where p-values for each individual variant is available, our method can be applied to the p-value sequence for the markers in a region (e.g. chromosome or gene) of interest to identify disease association. In addition, for whole genome or exome sequencing data, the marker density is likely to be higher and more variants may likely to be neutral. Our method has the potential to accommodate such data because it is less sensitive to the number of neutral variants than the other methods being compared with in this study and imposes no limit on the number of variants in the input sequence of interest.

Under the setting of smaller sample sizes such as 500 cases and 500 controls, the performance for the methods is worse than using larger sample sizes because rare variants would have been much harder to detect. As suggested by previous studies [41], a well-powered RVAS might aim for a discover sample of at least 15,000 cases for 50% power and 25,000 cases for 90% power, together with a substantial replication set given careful consideration of possible factors such as mutation rate, etc.

Our method uses permutations to obtain the empirical p-value. The permutation is carried out by shuffling the case–control label for the purpose of generating the empirical distribution for the general maximal segmental score while preserving the structure of the genotype data. This avoids distorting the structure of the genotype data so that the empirical distribution is drawn from permutation data with similar genotype structure to the original data. Under the simulation for the null hypothesis where there is linkage disequilibrium (LD) but no disease gene, the median number of SNPs for permutation-based segments is 66 and that for the observed segments is 60. The Q1 and Q2 are 29 and 124 SNPs for permutation segments, respectively. For the observed segments, the Q1 and Q2 are 26 and 139 SNPs, respectively. This result shows that the distribution of the permutation-based segments is similar to the observed. It suggests that the permutations do not result in a different LD pattern. The problem with cryptic population structure should be dealt with (such as using genomic control) prior to an association analysis as it indeed might produce false positive results.

For real data analysis, we use Parkinson’s disease case–control data as a model to demonstrate the application of GMSS. We confirm the association with SNCA and further identify disease association with HAS2 and KREMEN1. The permutation is not performed to correct for the number of comparisons. The p-values for SNCA and HAS2 are border-line. However, our method can be used as an alternative exploratory tool, not meant to replace other disease gene association methods.

In this study, we are only interested in testing the association between variants in a region, e.g., a chromosome or gene, and disease status. It is possible to apply the proposed method using genome-wide SNP data. There is no limitation for our method on the study design (e.g. family-based or case–control association studies) or the number of variants in the input sequence. However, our method is aimed to prioritize candidate regions and mitigate the problem with multiple-comparisons. In GWAS, the proportion of rare variants (<1%) is low. Applying our method to such data is feasible, however, it would not show the strength of our method which is designed to identify rare variant association and can be applied to data with both rare and common variants. For whole exome and whole genome sequencing studies, our method can be applied to a region (e.g. chromosome or gene) at a time and then adjust for multiple comparisons by the user’s method of choice as suggested by other methods for rare variant analysis [29].

The novelty of the current work is that we develop a different scoring system to improve the power for identifying rare-variant association. Our previous work is based on the context of common variant association [35,36] and its power for identifying rare variant association would be poor. The score system in the previous work uses discrete scores based on intervals of p-values. For GMSS, we develop a different scoring system to increase the power for identifying rare-variant association. In this method, the scores are continuous and the score system is a combination of chi-square statistic and a payoff term. Different from our previous work, the method proposed in this study aims to analyze data with a higher marker density and larger proportion of rare variants such as seen in TGP sequencing data. The simulations here are based on TGP data in order to mimic the structure of a more recent actual data. The method we propose in this study is to address the problems with identifying rare variant association as well as in situations where both rare and common variants contribute to disease risk.

## Conclusions

GMSS is a platform independent method that is time efficient and powerful for detecting rare variant association. It is capable of using information from both rare and common causal variants and requires no threshold for MAF or prior knowledge on candidate regions. GMSS is less sensitive to the inclusion of a large proportion of neutral variants and to the differences in direction of individual variant effects. Unlike other methods, the result from GMSS provides the p-value for disease association and a finer region of the input marker sequence for the association. Using Parkinson’s disease as a model, our method not only confirms association for a known gene but also identifies two genes previously found by other studies. In spite of many existing methods, we conclude that our method serves as an efficient alternative for exploring genomic data containing both rare and common variants to identify smaller regions within the data sequence most likely to be associated with the disease.

## Additional file

Additional file 1:
**A list of SNPs in the disease associated region identified by GMSS.**
