# Supplementary material for: Identifying rare and common disease associated variants in genomic data using Parkinson’s disease as a model
Source: J Biomed Sci. 2014 Aug 30;21(1):88. doi: 10.1186/s12929-014-0088-9 (PMC4428531; doi:10.1186/s12929-014-0088-9)
Supplement: Additional file 1: — A list of SNPs in the disease associated region identified by GMSS. [file 12929_2014_88_MOESM1_ESM.docx]

# Additional files

### Additional file 1 – A list of SNPs in the disease associated region identified by GMSS

| Chr | Gene | SNPs |
| --- | --- | --- |
| 4 | SNCA | rs10213062 rs13108925 rs12644119 rs17015992 rs3857047 rs17016018 rs17286290 rs3906628 rs6532186 rs356220 rs10003708 rs8180209 rs1045722 rs17016072 rs10024743 rs356168 rs2736990 rs356199 rs17016107 rs3796661 rs356192 rs3775439 rs2737029 rs10014396 rs12502363 rs3775442 rs3889917 rs1866995 rs894278 rs2119787 rs1442144 rs3822095 rs972880 rs1812923 rs10005233 rs11097238 rs2301134 rs2619363 rs2619364 rs1023777 rs10009760 rs12331318 rs17016279 rs2737026 rs6532194 rs6532196 rs10516849 rs1372513 rs11097239 rs6532197 rs1899389 rs6814481 rs6832819 rs1442140 rs1442138 rs10001067 rs1442137 rs1442136 rs1442134 rs1442132 rs3775464 rs3775465 rs10433954 rs10433955 rs3775467 rs9307077 rs10011790 rs10516850 rs10516851 rs3775478 rs41284765 rs2241884 SNP4-91075998 SNP4-91076502 rs10030931 rs3775483 rs1479429 rs1046994 rs13109927 rs12233759 rs11939062 rs13107488 rs17195014 rs6532210 rs13131903 rs10516854 rs13113259 rs11728457 rs1822527 rs6812321 rs17808648 rs17016459 rs12510783 rs13141923 rs724454 rs1443797 rs11929845 rs722937 rs722936 rs13114320 rs1443794 rs894803 rs2197291 rs34001515 rs7674520 rs12644131 rs17810668 rs6837278 rs1835522 rs17016596 rs10516866 rs10461168 rs13109951 rs9884304 rs17008807 rs2116123 rs4356886 rs10015971 rs17016697 rs6832140 rs10013360 rs11943050 rs11945084 rs28812473 rs7676938 rs7684334 rs1350855 rs11946195 rs11937852 rs13147804 rs10516867 rs10024732 rs1037926 rs12647859 rs2870246 rs12330928 rs2169399 rs17016737 rs11723677 rs17227797 rs10015182 rs11941084 rs7693233 rs17016824 rs12642077 rs8180269 rs17016859 rs4693236 rs17016910 rs17016912 rs6842950 rs17016931 rs1903577 rs7688033 rs6847189 |
| 8 | HAS2 | rs13256476 rs13267240 rs12547273 rs10956026 rs7816365 rs7834611 rs17276050 rs16895122 rs975037 rs2046571 rs2129536 rs16895166 rs1386443 rs13257411 rs13255877 rs16895174 rs7825695 rs10099580 rs10505400 rs6987154 rs6415475 rs16895201 |
| 22 | KREMEN1 | rs9625679 rs134557 rs134558 rs134559 rs16987014 rs134560 rs760628 rs134583 rs134585 rs134592 rs134594 rs134609 rs134615 rs134651 rs7289560 rs10483154 rs2205771 rs12628787 rs5752866 rs8141812 rs8138320 rs126077 rs12158320 rs5752867 rs132268 |
